# The impact of psychological resilience on chronic patients’ depression during the dynamic Zero-COVID policy: the mediating role of stigma and the moderating role of sleep quality

**DOI:** 10.1186/s40359-023-01248-6

**Published:** 2023-07-21

**Authors:** Yujin Mei, Xue Yang, Changjun Liu, Yuqing Li, Jiaofeng Gui, Lin Zhang

**Affiliations:** 1 School of Nursing, Wannan Medical College, 22 Wenchang West Road, Higher Education Park, Wuhu City, Anhui Province P.R. China; 2 School of Marxism, Jinzhou Medical University, No.40, Section 3, Songpo Road, Linghe District, Jinzhou City, Liaoning Province P.R. China; 3 Department of Internal Medicine Nursing, School of Nursing, Wannan Medical College, 22 Wenchang West Road, Higher Education Park, Wuhu City, An Hui Province P.R. China

**Keywords:** Psychological resilience, Stigma, Sleep, Depression, Chronic patients

## Abstract

**Objective:**

Chronic patients are experiencing depression caused by themselves or the surrounding environment, how to cope with the change of mentality and adjust the psychological stress response, especially under the background of the current dynamic Zero-COVID policy in China, is a problem worth further discussion. The researchers constructed a mediating regulation model to test the influence of psychological resilience on depression of chronic patients during dynamic Zero-COVID, as well as the mediating role of stigma and the moderating role of sleep.

**Method:**

From October 2021 to February 2022, this study used a multi-stage sampling method and random number table method to collect data in the Shang Cheng District of Hangzhou City, Zhejiang Province. Firstly, the Second Affiliated Hospital of the Zhejiang University School of Medicine, a third-class hospital was randomly selected from the Shang Cheng District. Secondly, three departments were strategically selected from this hospital: endocrinology, dermatology, and traditional Chinese medicine. Thirdly, survey points were set up in each department, and chronic patients were strategically selected for questionnaire surveys. Finally, a face-to-face survey was conducted on 398 chronic patients who met the criteria for inclusion. In addition, chronic medical illness burden was assessed using the Cumulative Illness Rating Scale-Geriatrics (CIRS-G), psychological resilience was measured by the Conner-Davidson Resilience Scale (CD-RISC), stigma was measured by the Stigma Scale for Chronic Illness (SSCI), sleep was measured by the Pittsburgh Sleep Quality Index(PSQI) and depression was estimated by the Patient Health Questionaire-9(PHQ-9). SPSS (version 25.0) and PROCESS (version 4.0) were used for correlation analysis, mediation analysis, and mediated moderation analysis.

**Results:**

Psychological resilience was negatively correlated with depression, stigma, and sleep. Depression was positively correlated with stigma and sleep. Stigma and sleep were positively correlated; Stigma played a mediating role in the relationship between psychological resilience and depression; Sleep moderated the first half of the pathway “psychological resilience $\to$ stigma $\to$ depression”.

**Conclusion:**

Psychological resilience affected depression directly and indirectly through stigma. At the same time, sleep played a moderating role between psychological resilience and depression. The correlation between psychological resilience and stigma was stronger when levels of sleep levels were higher.

## Introduction

COVID-19 is a highly infectious disease that spreads rapidly. It has caused grievous health threats all over the world [1]. Although China had successfully controlled the epidemic in the past two years, its suddenness and high transmission among people still posed an enormous threat and impact on people’s lives. To further strengthen epidemic prevention and control of COVID-19, the China COVID-19 epidemic prevention and control headquarters planned a " dynamic zero-COVID " policy on Dec 7, 2021 to reduce personnel mobility and the possibility of another outbreak of the epidemic [2].

Chronic diseases, also known as chronic non-communicable diseases, are a general term for a group of non-communicable diseases with a long course of disease, complex causes and difficult to cure once they become ill, and the main common chronic diseases are cardiovascular diseases (hypertension and coronary heart disease), cancer, diabetes, and chronic respiratory diseases (bronchial asthma) [3]. Chronic disease is increasingly recognized as a serious, worldwide public health concern. Every year, 41 million people die from chronic diseases, which is equivalent to 71% of all deaths globally [4]. In China, the prevalence of chronic diseases is increasing. A report from a nationwide survey showed that more than 20% of people aged 18 years and above suffer from at least one chronic disease [3]. More concerning, chronic diseases continue to become the predominant disease burden [5]. Chronic diseases were generally considered incurable, which was considered one of the stress factors for chronic patients [6]. Previous studies had shown that in addition to treatment-related problems, chronic patients also faced many psychological pressures [7]. Moreover, because the treatment of this disease was a long-term process and accompanied by complications, it affected the psychosocial function of patients [8]. Patients would have many mental diseases, such as stress, depression and anxiety, and depression was most common in chronic patients [9]. Since the hospital had a large flow of people with unknown contact history, and with the formulation of the dynamic clearance policy, chronic patients experienced additive psychosocial pressure [10]. It had been reported that some patients were seriously affected in their daily lives by excessive stress [11]. In addition to long-term treatment, the outbreak of covid-19, lack of understanding of chronic diseases and commuting to and from the hospital make patients face prominent contradictions in dealing with chronic diseases in the pandemic [12]. This fact may lead to an increase in depression rate in chronic patients and then affect the prognosis of the disease. Therefore, it is important to examine how depression is affected in chronic patients in the context of the dynamic Zero-COVID policy.

Psychological resilience (PR) is defined as the ability to actively respond to stressful events, including adaptation to the environment and positive adaptation after adversity [13]. Unlike other mental health indicators, psychological resilience was a dynamic process that affected individuals in psychology, behavior, and physiology [14]. Previous evidence showed that social support is a core component of resilience and was considered a predictor of depression [3, 15]. The concept of an upbeat coping style assumes that older people with higher PR participate in more preventive health behaviors, report more social participation organizations, and have a higher level of mental health [16, 17]. On the contrary, for patients with chronic diseases who lack social support and optimism, the PR level is lower than that of the average population, and they may be more vulnerable to the adverse impact of mood [18]. These findings are related to the biology, psychology, and social psychology of depression. Meanwhile, another study pointed out that PR as a protective factor could reduce the incidence of depression to some extent [19]. Therefore, exploring the relationship between PR and depression in chronic patients is of great significance.

### The mediating role of stigma

Stigma is manifested in social prejudice and exclusion of patients with specific diseases [20]. Patients’ internalization of this discrimination may affect their psychological state and quality of life [21]. As the age of the disease increases, most patients with chronic diseases will experience more stigma, including avoiding social interaction, refusing to communicate, and passively coping with the illness [22]. In recent years, stigma among patients with chronic diseases has been increasing, affecting their physical and mental status and quality of life [23]. At the same time, high levels of stigma can lead to negative experiences for people with chronic diseases and affect their mental health [24]. With the growth of age, the incidence of complications in patients with chronic diseases tends to increase, and the occurrence of complications increases the stigma of patients to a certain extent, leading to a higher degree of depression [25, 26]. At the same time, stigma may lead to daily communication difficulties and loss of social skills [27]. Many studies have shown that compared with the subjects in the standard group, the patients with stigma show a significantly higher tendency to depression [28, 29]. Therefore, low-level stigma is an essential factor in improving depression.

Psychological and neuroscience research showed that emotions were related to mental health. Negative emotions are common in clinical and chronic diseases, which are usually a precursor to the decline of psychological quality [30, 31]. Research showed that PR had a much more substantial impact on emotion than emotion does on PR [32], which means that PR was the driving factor of emotional change. As a negative emotion, stigma affects the level of individual PR and plays an important role in the construction of individual PR. Previous cross-sectional studies have confirmed the negative predictive effect of PR on stigma, which is significantly higher in patients with low stigma than in those with high stigma [33, 34]. High and low levels of PR have beneficial and harmful effects on various emotional and physical outcomes, respectively [35]. Specifically, a higher PR level is related to a more positive coping style, a more optimistic attitude, and executive ability. PR is usually considered a potential psychological factor, and chronic patients exposed to negative emotions have a worse healthy psychological state than those without negative emotions [36]. Therefore, we formulated the hypothesis 1 : stigma as a negative emotion may play an intermediary role between PR and depression.

### The moderating role of sleep

Although PR may affect depression through the mediation of stigma, not all older adults with high levels of PR report low levels of stigma and lower levels of depression, the heterogeneity of the results may depend on the individual characteristics that regulate the effects of PR on stigma and depression. Sleep was an essential physiological process with a vital recovery function [37]. Poor sleep quality would affect lifestyle changes and increase the risk of falls and disease status of chronic patients. Sleep disorders could also lead to cognitive dysfunction, mainly manifested in memory defects, inattention, slow response, etc. Sleep disorders pose serious health problems for chronic patients, increasing the risk of depression, suicidal ideation, health behavior, and mortality [38, 39]. Consistent with this concept, previous studies had confirmed that sleep disorders were significantly negatively correlated with PR [40]. Psychological factors, such as depression, could worsen poor sleep quality [41].

At the same time, sleep quality is significantly related to mental health. Many cross-sectional studies had shown that sleep quality was positively correlated with various negative emotions [42, 43]. Although it was unclear whether PR directly predicts sleep quality, many studies reported that chronic patients with low-level PR were more vulnerable to the adverse effects of poor sleep quality during the influenza pandemic, which reveals the critical link between PR and sleep quality [44]. In addition, stigma was considered a common obstacle to seeking treatment in the research on the determinants of help-seeking behavior of insomnia patients [45]. Specifically, studies had shown that people with sleep quality problems might have a stricter sense of shame [46]. So, we formulated hypothesized 2 : sleep quality might moderate the relationship between PR and stigma.

### The present study

This study proposes a moderated mediation model to investigate the direct/indirect relationship between PR and depression in chronic patients. Specifically, this study suggested the following hypotheses: (H1) PR is negatively correlated with depression, PR is negatively associated with stigma, stigma is positively correlated with depression, and stigma mediates the relationship between PR and depression; (H2) Sleep quality moderated the pathways between PR and stigma. In addition, depression will change as a function of sleep quality.

## Materials and methods

### Participants and data collection

From October 2021 to February 2022, this study used a multi-stage sampling method and random number table method to collect data in the Shang Cheng District of Hangzhou City, Zhejiang Province. Firstly, the Second Affiliated Hospital of the Zhejiang University School of Medicine, a third-class hospital was randomly selected from the Shang Cheng District. Secondly, three departments were strategically selected from this hospital: endocrinology, dermatology, and traditional Chinese medicine. Thirdly, survey points were set up in each department, and chronic patients were strategically selected for questionnaire surveys. Finally, a face-to-face survey was conducted on 398 chronic patients who met the criteria for inclusion. The questionnaires in this study were asked and completed face-to-face and one-to-one by uniformly trained investigators. 398 questionnaires were distributed and 370 valid questionnaires were collected after excluding missing and invalid questionnaires, with a valid return rate of 92.96%. The inclusion criteria used for the participants were as follows: (i) met diagnostic criteria for chronic illness and age ≥ 45 years; (ii) good verbal skills and no barriers to communication with the investigator; (iii) informed about the diagnosis and accepted this study. Exclusion criteria: (i) patients with serious psychiatric disorders who are unable to communicate and express themselves normally; (ii) patients who did not cooperate with this investigation.

To reduce errors, seventeen investigators were uniformly trained before the survey to clarify communication skills and scoring criteria. After obtaining informed consent from patients with chronic diseases, questionnaires were distributed and asked one-to-one by investigators, who then filled out the questionnaires. All methods are implemented following the declaration of Helsinki.

### Measurements

#### Chronic medical illness burden

The Cumulative Illness Rating Scale-Geriatrics (CIRS-G) was an organ-system based rating scale, with the most severe condition occurring in each of 14 organ systems assigned a severity score from 0 (no problem) to 4 (extremely severe), resulting in a final score ranging from 0 to 56 [47]. The CIRS-G severity index was calculated by dividing the total CIRS-G score by the number of organ systems endorsed in the CIRS-G and provided an estimate of the overall severity of dysfunction. The Cronbach’s alpha of the scale in this study was 0.831, and the KMO measure was 0.810 (*P* < 0.01).

#### Psychological resilience

American psychologist Connor and Professor Davidson compiled the conner-Davidson Resilience Scale (CD-RISC) in 2003 [48]. The CD-RISC contains 25 items, which were rated on a five-point Likert scale and range from 0 (“Not true at all”) to 4 (“True nearly all the time”). Possible scores thus range from 0 to 100. These items could correspond to the five-factor. The first factor reflected high standards, tenacity, and competence (eight items). The second factor reflected handling negative emotions, trusting one’s instincts, and perceived benefits of stress (seven items). The third factor reflected positive attitude to change and secure relationships (five items). The fourth one reflected perceived control (three items), and the fifth one was spirituality (two items). The Cronbach’s alpha of the scale in this study was 0.861, and the KMO measure was 0.713 (*P* < 0.01).

#### Stigma

The Stigma Scale for Chronic Illness (SSCI) was developed by Rao to measure stigma experienced by individuals with chronic neurological disorders, included stroke [49]. It consisted of 24 items and contains two subscales: felt stigma and enacted stigma. The first 13 items refer to the felt stigma, asked questions about the respondent’s feelings. The following eleven items referred to the enacted stigma, asked questions about the behavior of others towards the respondent. Each item was rated on a scale from 0 (never) to 4 (always). A higher score indicated a higher frequency of experiencing stigma. The Cronbach’s alpha of the scale in this study was 0.829, and the KMO measure was 0.864 (*P* < 0.01).

#### Sleep

The Pittsburgh Sleep Quality Index (PSQI) was developed by Buysse et al. [50]. The index had seven components: subjective sleep quality, sleep latency, duration, habitual sleep efficiency, sleep disorders, use of sleep drugs, and daytime dysfunction in the previous month. The score range was 4 points, ranging from 0 (none) to 3 (≥ 3 times a week). In China, a score ≥ 7 had high diagnostic sensitivity and specificity in distinguishing patients with poor sleep from healthy subjects. The total score of PSQI ranged from 0 to 21. The higher the score, the worse sleep quality. The Cronbach’s alpha of the scale in this study was 0.730, and the measured value of KMO was 0.700 (*P* < 0.01).

#### Depression

The Patient Health Questionaire-9 (PHQ-9) was derived from the depression part in the Patient Health Questionnaire (PHQ) compiled by Spitzer in 1999 [51]. The response options for the project ranged from “not at all” (0 points) to “almost every day” (3 points). The scale could not only screen for depression but also show the severity of depression. Because of its convenient use, good reliability, and effectiveness, it had been widely used in grass-roots hospitals. The Cronbach’s alpha of the scale in this study was 0.842, and the measured value of KMO was 0.583 (*P* < 0.01).

### Statistical analyses

SPSS25.0 was used to conduct all statistical analyses. Descriptive analyses, independent sample T-test, and one-way analysis of variance (ANOVA) were used to describe demographic characteristics and the significance of depression among chronic patients with different features, respectively. Pearson correlation coefficients described the relationship between the four variables (PR, stigma, sleep, depression).

Furthermore, a mediation model was set up with PR as the independent variable, stigma as the mediating variable, depression as the dependent variable, and age, gender, and education level as control variables. The SPSS PROCESS 4.0 macro (Model 4) was conducted to test the mediating effect of stigma, and 95% confidence intervals (CIs) were calculated by adopting a bias-corrected non-parametric percentile bootstrapping method with 5000 samples. We established a moderated mediation model with sleep as a moderating variable based on the mediation model. The SPSS PROCESS 4.0 macro (Model 59) was conducted to test the moderating effect of sleep. Simple slope tests were used to describe the relationship between PR and morbidity stigma under different sleep.

## Results

### Descriptive statistics

398 questionnaires were distributed and 370 valid questionnaires were collected after excluding missing and invalid questionnaires, with a valid return rate of 92.96%. Table 1 showed the demographic characteristics of the study objects and a Univariate analysis of depression with different features. Of the 370 chronic patients, 230 (62.2%) were men, and 140 (37.8%) were women. The age range of chronic patients was 45~95 years, with an average age of 63.71 ± 10.47 years. Most study participants (68.6%) reported middle school education or lower. At least 51.3% of the respondents live in the city. There were differences between the depression in marital status, economic situation, cigarette smoking, alcohol use experience, and physical exercises habit.

**Table 1:** Univariate analysis of depression of chronic patients with different characteristics (N = 370)

Variables	Group	N (%)	Mean ± SD	*F/t*	*P*
Gender	Male	230(62.2)	6.66 ± 4.12	1.713	0.191
	Female	140(37.8)	7.28 ± 4.90		
Age	45 ~ 64	204(55.1)	6.57 ± 4.90	1.332	0.265
	64 ~ 74	122(33)	7.16 ± 3.53		
	≥ 75	44(11.9)	7.62 ± 4.39		
Education lever	Middle school or less	254(68.6)	6.87 ± 4.50	0.403	0.669
	High school or technical secondary school	64(17.3)	7.24 ± 4.41		
	Junior college or university	52(14.1)	6.48 ± 4.15		
Marital status	Married	355(4.1)	6.80 ± 4.41	4.036	0.045
	Single	15(95.9)	9.21 ± 4.51		
Economic situation	Live beyond one’s income	49(13.2)	7.67 ± 4.92	3.377	0.035
	Balance of payments	165(44.6)	7.31 ± 4.81		
	In credit	156(42.2)	6.21 ± 3.74		
Residence	Rural	40(39.7)	6.89 ± 4.69	0.030	0.971
	Suburb	35(9.5)	7.06 ± 4.76		
	Urban	188(50.8)	6.86 ± 4.19		
Medical insurance	No	18(4.9)	8.42 ± 2.63	1.063	0.346
	Medical or other insurance	330(89.2)	6.79 ± 4.52		
	Free medical care	22(5.9)	7.38 ± 3.98		
Cigarette smoking	No	208(56.2)	7.41 ± 4.92	3.386	0.035
	Ever smoking	115(31.1)	6.31 ± 3.55		
	Current smoking	47(12.7)	6.01 ± 3.79		
Alcohol use experience	No	211(57)	7.59 ± 4.84	6.833	0.001
	Ever drinking	82(22.2)	6.30 ± 3.65		
	Current drinking	77(20.8)	5.60 ± 3.61		
Physical exercises habit	No	55(14.8)	9.54 ± 4.78	13.663	< 0.001
	Less than regular physical exercises	213(57.6)	6.82 ± 4.45		
	Regular physical exercises	102(27.6)	5.71 ± 3.64		

### Disease status of the patient

The scores for CIRS-G were 4.59 ± 2.97. As shown in Table 2, the five most common disease types were respiratory (18.9%), nervous system (17.3%), vascular (16.2%), cardiac (12.7%) and endocrine/metabolic and breast cancer (11.9%) diseases.

**Table 2:** Disease status of the patient (N = 370)

Variables	N (%)
Cardiac	47 (12.7)
Vascular	60(16.2)
Hematopoietic	17(4.6)
Respiratory	70(18.9)
Eye/Ear/Nose/Throat	12(3.2)
Upper Gastrointestinal	24(6.5)
Lower gastrointestinal	43(11.6)
Hepatic	38(10.3)
Renal	22(5.9)
Genitourinary	17(4.6)
Musculoskeletal	38(10.3)
Nervous system	64(17.3)
Endocrine/Metabolic and Breast cancer	44(11.9)
Psychosis	22(5.9)

### Bivariate correlation analyses

Table 3 showed the correlational analyses between the study variables. PR was negatively correlated with stigma (*r* = − 0.168, *P* < 0.01) and depression (*r* = − 0.352, *P* < 0.01). Stigma was positively correlated with depression (*r* = 0.605, *P* < 0.01) and sleep (*r* = 0.398, *P* < 0.01). Sleep was negatively correlated with PR (*r* = − 0.234, *P* < 0.01), but was positively correlated with depression (*r* = 0.398, *P* < 0.01). The scores for PR, sleep , stigma, and depression were 50.25 ± 14.05, 44.09 ± 15.44, 6.50 ± 3.33, and 6.89 ± 3.43, respectively.

**Table 3:** Descriptive statistics and correlations among variables (N = 370)

Variables	Mean	SD	1	2	3	4
1 PR	50.25	14.05	─			
2 Stigma	6.50	3.33	−0.168^***^	—		
3 Sleep	44.09	15.44	−0.234^***^	0.398^***^	—	
4 Depression	6.890	3.43	−0.352^***^	0.605^***^	0.398^***^	—

PR, psychological resilience; ***p < 0.001

### The mediation analyses

To examine hypothesis 1, we examined the mediating role of stigma on the relationship between PR and depression with PROCESS 4.0 macro (Model 4) proposed by Hayes after controlling for the demographic variables of age, gender, and education level (Table 4). It showed that PR was negatively associated with depression (*β* = −0.087, *P* < 0.001). PR was negatively associated with stigma (β = −0.172, *P* < 0.001), and PR explained the total of 3.70% of the stigma (F = 3.475, *P* < 0.001, ΔR² = 0.037). In addition, the result showed that stigma was positively associated with depression (*β* = 0.162, *P* < 0.001), and the explanation of PR for depression increased to 43.9% (F = 56.955, *P* < 0.001, ΔR² = 0.439).

**Table 4:** Testing the mediation effect of psychological resilience on depression

Predictors	On Stigma				On Depression			
	*β*	*SE*	*t*	95%*CI*	*β*	*SE*	*t*	95%*CI*
Age	0.521	1.148	0.454	−1.735,2.778	0.280	0.252	1.113	−0.215,0.776
Gender	2.793	1.641	1.702	−0.434,6.020	−0.063	0.361	−0.174	−0.774,0.648
Education level	−0.427	1.130	-0.378	−2.649,1.795	0.501	0.248	2.021	0.014,0.989
PR	−0.172	0.059	−2.937^**^	−0.288, − 0.057	−0.087	0.013	−6.677^***^	−0.113, − 0.061
Stigma					0.162	0.011	14.090^***^	0.139,0.184
*R* ^*2*^	0.037				0.439			
*F*	3.475				56.955			

Analyses conducted using PROCESS Model 4; PR, psychological resilience; **p < 0.01, ***p < 0.01

We examined the direct effect (B = − 0.087, SE = 0.013, 95%CI = [− 0.113, − 0.063]) and indirect effects (B = − 0.028, SE = 0.011, 95%CI = [− 0.049, − 0.007]) of PR on depression by testing 95% confidence intervals (CIs) based on 5000 bootstrapped samples, indicating stigma partially mediated the relationship between PR and depression (Table 5). The mediating and direct effects accounted for 24.35% and 75.65% of the total effect, respectively.

**Table 5:** Total effect, direct effect and indirect effect among the variables

	Effect	BootSE	BootLLCI	BootULCI	Relative effect size
Indirect effect	−0.028	0.011	−0.049	−0.007	24.35%
Direct effect	−0.087	0.013	−0.113	−0.063	75.65%
Total effect	−0.115	0.016	−0.146	−0.083	100.00%

### The moderation analyses

To examine hypothesis 2, we adopt the PROCESS macro (Model 59) proposed by Hayes to examine the moderated mediation. Specially, we estimated parameters for two models. On stigma, we evaluated the moderating effect of sleep quality on the relationship between PR and stigma. On depression, we estimated the moderating impact of sleep quality on the relationship between PR and stigma, and the relationship between stigma and depression. If the 95%CI interval did not include 0, it indicated that there was a moderating effect (*P* < 0.05), and vice versa.

**Fig. 1:**
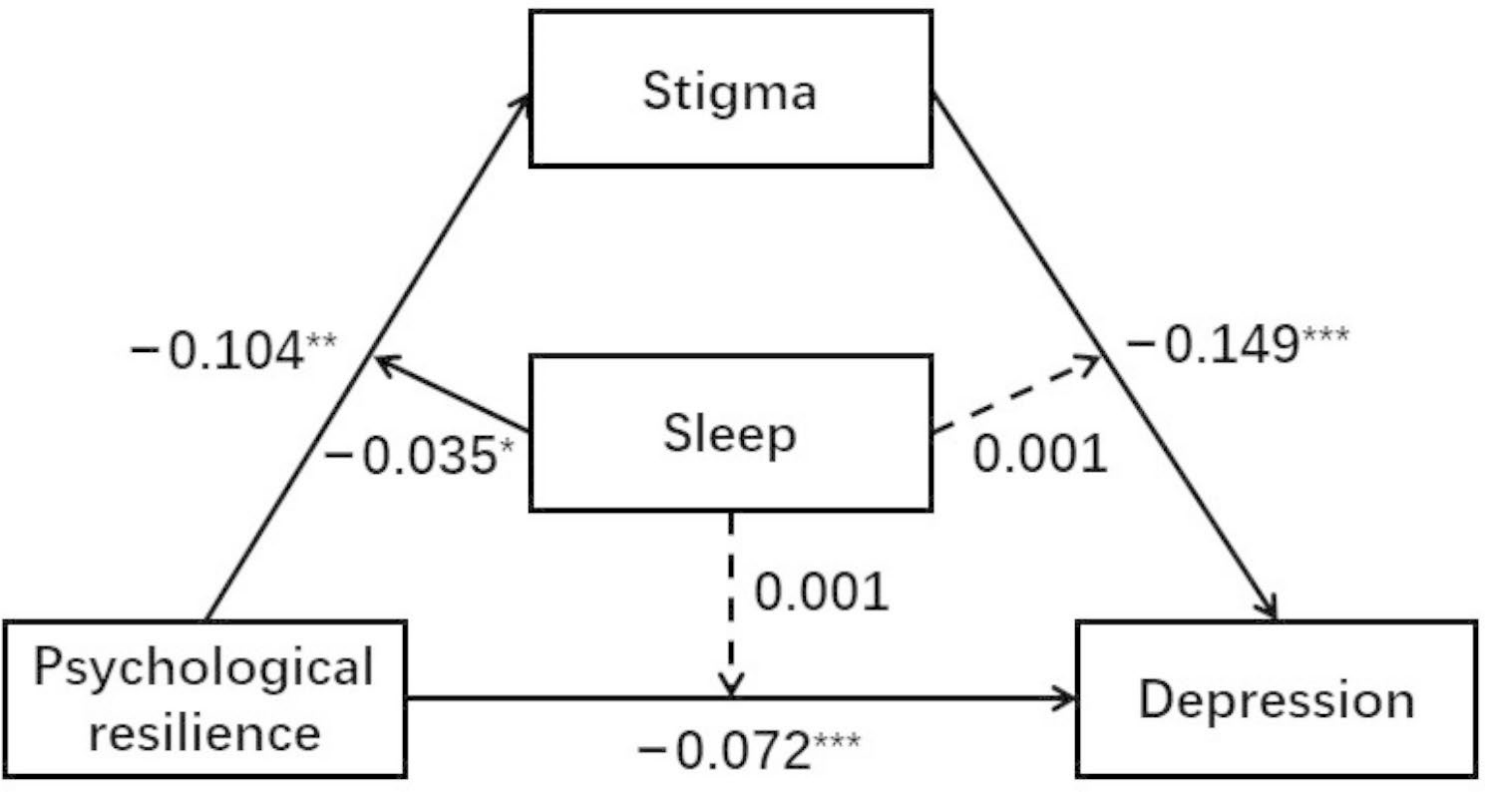
The moderated mediation model. *p < 0.05, **p < 0.01, ***p < 0.001

As shown in Table 6, Model 1 revealed a major impact of PR on stigma (B = 0.125, SE = 0.121, 95%CI = [− 0.114, 0.364]), which was moderated by sleep quality (B = − 0.035, SE = 0.016, 95%CI = [− 0.066, − 0.005]). Model 2 revealed a major impact of PR on depression (B = − 0.077, SE = 0.273, 95%CI = [0.005, − 1.151]), which was not moderated by sleep quality (B = 0.001, SE = 0.004, 95%CI = [− 0.006, 0.008]). In addition, it revealed a major impact of stigma on depression (B = 0.139, SE = 0.023, 95%CI = [0.095, 0.184]), which was not moderated by sleep quality (B = 0.002, SE = 0.003, 95%CI = [− 0.004, 0.007]). Hence, hypothesis 2 was partially supported. The final moderate mediation model was shown in Fig. 1.

**Table 6:** Results of the moderated mediation model analysis

Predictor	Model 1(Stigma)				Model 2( Depression)			
	*B*	*SE*	*t*	95%*CI*	*B*	*SE*	*t*	95%*CI*
Age	0.386	1.121	0.344	−1.819,2.591	0.204	0.244	0.838	−0.275,0.684
Gender	1.990	1.608	1.237	−1.172,5.151	−0.227	0.351	−0.648	−0.917,0.463
Education lever	−1.232	1.119	−1.120	−3.433,0.969	0.382	0.244	1.567	−0.097,0.862
PR	0.125	0.121	1.032	−0.114,0.364	−0.077	0.273	−2.817^**^	0.005, − 1.151
Sleep	2.631	0.768	3.426^***^	1.121,4.140	0.182	0.257	0.709	−0.323,0.687
Stigma					0.139	0.023	6.169^***^	0.095,0.184
PR×Sleep	−0.035	0.016	−2.259^*^	−0.066, − 0.005	0.001	0.004	0.225	−0.006,0.008
Stigma×Sleep					0.002	0.003	0.508	−0.004,0.007
*R* ^*2*^	0.091				0.481			
*F*	6.067				41.843			

Analyses conducted using PROCESS model 59, PR: psychological resilience. *p < 0.05, **p < 0.01, ***p < 0.001

Model 1: Adjusted for age, gender, education lever, PR, sleep and PR×Sleep.

Model 2: Adjusted for age, gender, education lever, PR, sleep, stigma, PR×Sleep and Stigma×Sleep

**Fig. 2:**
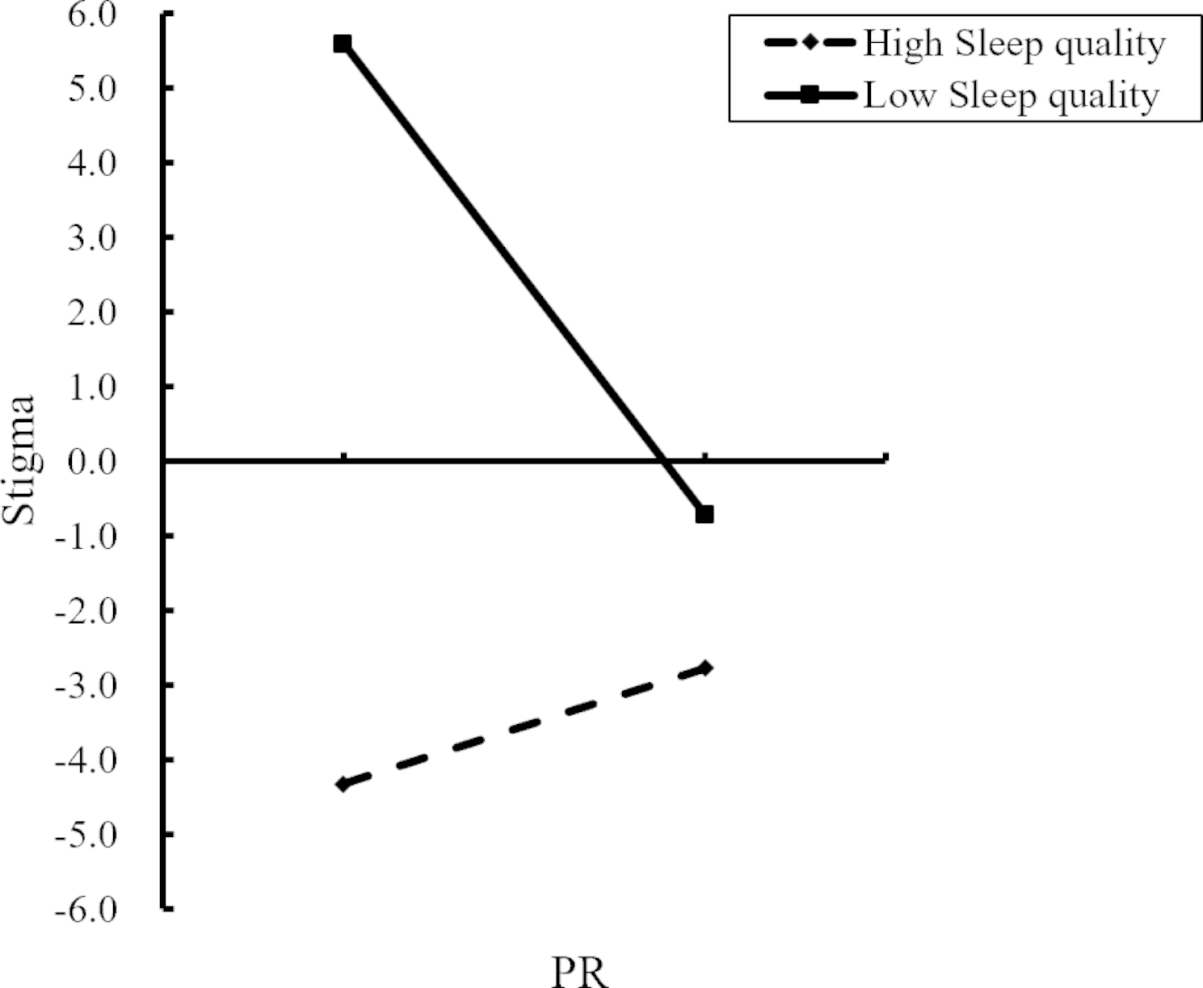
The interaction effect of psychological resilience and sleep on stigma

In order to examine the direction and trend of the moderating effect of sleep quality on PR and stigma, the relationship between PR and stigma was analyzed at three levels of sleep quality: good (M-1SD), moderate (M) and poor (M + 1SD), according to the mean and mean plus or minus one standard deviation, as shown in Table 7. Confidence intervals contained 0 when patients with chronic illness had high sleep quality, indicating that PR was not a predictor of stigma; and confidence intervals did not contain 0 when patients had low sleep quality, indicating that PR was a significant predictor of sickness stigma, as shown in Fig. 2.

**Table 7:** Conditional indirect effects of sleep on stigma

Sleep	Effect	BootSE	BootLLCI	BootULCI
M − 1SD	−0.003	0.015	−0.034	0.026
M	−0.020	0.009	−0.039	−0.002
M + 1SD	−0.038	0.013	−0.064	−0.012

## Discussion

The present study constructed a moderated mediation model to investigate whether PR was indirectly associated with depression through stigma and whether sleep moderated the direct relationship between PR and stigma. The findings showed that stigma partially explained the effect of PR on depression. This indirect relationship was moderated by sleep in the one-stage path. Low sleep quality exacerbates PR’s impact on stigma, whereas this effect was not significant among chronic patients with high sleep quality. This study was the first to explore the mechanism of PR and depression, which provided theoretical foundations and directions for promoting depression among chronic patients.

### Mediating role of stigma

The current results showed that chronic patients with higher PR levels were more likely to report lower levels of depression, which was similar to previous studies [52]. Many mental health studies believe that positive and negative PR was ultimately applied to the self and transformed into the impact on personal emotions and behaviors [53, 54]. From the psychological and behavioral development perspective, emotions affected the individual’s cognitive and behavioral structure [55]. Many studies showed that chronic patients with lower-level PR were less likely to engage in healthy lifestyles, which led to an increased risk of death [56]. Compared with positive emotions, negative emotions had a much more significant impact on the essential behavioral outcomes of patients. In addition, research showed that low-level PR weakened the beneficial effect of high-level PR on happiness [57]. Therefore, we should consider improving patients’ depression by increasing PR levels in chronic patients.

Our findings also indicated that stigma mediated the relationship between PR and depression in chronic patients. Based on the conceptual model of stress theory, when chronic patients faced negative emotions, if they could actively call internal coping resources and seek external social help, it can be transformed into active behavioral adaptation, and it was still possible to maintain a good quality of life and mental health [58, 59]. It shows that chronic patients with low-level PR can reduced their stigma through reasonable coping methods to minimize the damage caused by stigma and slow down the process of depression [60]. Specifically, as a harmful negative emotion, stigma could activate the autonomic nervous system, neuroendocrine, metabolic, and immune systems while reducing the activities of the parasympathetic nervous system, leading to adverse physical and psychological conditions [61, 62]. In addition, long-term exposure to high levels of stigma and low levels of PR can lead to chronic disease patients losing the ability to live independently and in social contact [63]. Numerous longitudinal study has also shown that negative emotion was associated with a greater level of physical frailty and an increased risk of cardiovascular disease [64], which accelerated the development of depression.

### Moderating role of sleep

This study examined the moderating role of sleep as a moderating variable in the model. It was found that sleep moderated the first half of the mediating pathway of the model, sleep moderated the relationship between PR and stigma. Specifically, PR was only associated with stigma in chronic patients with who had low sleep quality, and this relationship was not significant in chronic patients who had high sleep quality. A study showed that people with high sleep quality better cope with trauma and stress [65]. Stress would cause hormone level disorder and body function decline, increasing the probability of complications in patients [66]. However, complications are one of the essential mechanisms for developing stigma in chronic patients [67]. Therefore, sleep quality is closely related to the level of stigma. In addition, after controlling the covariates, chronic patients with low sleep quality showed a decreased sense of participation, resulting in an increased risk of depression.

In contrast, high sleep quality helped improve PR and mental health by reducing negative emotions [68, 69]. Therefore, high-quality sleep seems to be a protective factor, which could enhance the harmful effect of low-level PR on the stigma of chronic patients. Thus, to improve the depression of chronic patients, it was necessary to focus not only on patients’ psychological resources and call ability and strive to develop their psychological resilience but also on patients’ sleep quality.

### Limitations

This study had several limitations worth considering. Firstly, this study was cross-sectional, and the causal relationship between PR, stigma, sleep, and depression cannot be inferred. A longitudinal examination should be conducted further to reveal the causal relationship between PR and depression. Secondly, although some potential confounding factors were controlled by us (such as age, gender, and education level), other confounding variables were not maintained. Future research should add more covariates to reduce the interference of effect estimation. Thirdly, the respondents were only chronic patients in one hospital, which was not representative. In the future, random sampling should be used to investigate the population of different hospitals in different regions. They should be grouped into different regional groups to explore the changes in each group. In addition, other mediating variables could be introduced to examined the impact mechanism of depression further and provide new goals for formulating measures to improve depression in chronic patients.

### Implications

Theoretically, the current research is conducive to a deeper understanding of the mechanism of PR affecting depression and clarifies the relationship between PR, stigma, sleep, and depression. In a practical sense, this study can provide a new perspective for chronic patients to improve their depression, and family members and medical staff should pay attention to it. Since low-level PR and stigma have been identified as the main risk factors for increasing the depression rate of chronic patients, we emphasize the clinical importance of providing psychological help and mental health education. In addition, high-quality sleep may be related to stigma and depression. We should prioritize the prevention and intervention of chronic disease patients with low-level PR and poor sleep quality, which will help delay the onset of complications and reduce the possibility of depression.

## Conclusion

It is the first time we have established a mediation model between PR and depression. Our findings suggested that stigma played a mediating role in the association between PR and depression. In addition, sleep regulated the relationship between PR and stigma. However, this modernization effect was only significant in chronic patients with high-quality sleep. In other words, when chronic patients had lower quality sleep, the impact of PR on a stigma would increase. Therefore, we should fully understand the relevant information about chronic patients, screen chronic patients with psychological abnormalities, and carry out mindfulness interventions to improve low-level pr. At the same time, medical staff should regularly give public lectures on the prevention and control measures of stigma and depression to achieve early screening, diagnosis, and intervention. In addition, the community should provide high-quality leisure and entertainment places for chronic patients, and actively organize cultural and artistic performances such as square dance, so that chronic patients can cultivate their sentiment in entertainment, reduce their sense of stigma, and improve their depression.

## Data Availability

The datasets used and analyzed during the current study are available from the corresponding author on reasonable request.

## Abbreviations

ANOVA: Analysis of variance
B: Unstandardized
CD-RISC: Conner-Davidson Resilience Scale
CI: Confidence interval
CIRS-G: Cumulative Illness Rating Scale-Geriatrics
KMO: Kaiser Meyer Olkin
MMSE: Mini-Mental State Examination
PHQ-9: Patient Health Questionaire-9
PR: Psychological resilience
PSQI: Pittsburgh Sleep Quality Index
SD: Standard deviation
SE: Standard error
SSCI: Stigma scale for Chronic Illness
WHO: World Health Organization

## References

[1] Zhang JJ, Dong X, Liu GH (2023). Risk and protective factors for COVID-19 morbidity, severity, and Mortality[J]. Clin Rev Allergy Immunol.

[2] Bai W, Sha S, Cheung T (2022). Optimizing the dynamic zero-COVID policy in China[J]. Int J Biol Sci.

[3] The State Council Information Office. The State Council Information Office held a press conference on the. “Report on the Nutrition and Chronic Disease Status of Chinese Residents. (2020). Available online at: http://www.gov.cn/xinwen/2020-12/24/content_5572983.htm (accessed December 12, 2020).

[4] World Health Organization. Noncommunicable Diseases. WHO. (2021). Available online at: https://www.who.int/news-room/fact-sheets/detail/noncommunicable-diseases (accessed October 11, 2022).

[5] Chinese Center for Disease Control and Prevention (2020). Chinese National Death cause Surveillance dataset.

[6] John PA, Martin TW, Janey CP (2019). Interventions to support behavioral self-management of ChronicDiseases[J]. Annu Rev Public Health.

[7] Emanuele F, Chiara F, Stefania A et al. Psychological and emotional impact of COVID-19 pandemic on people living with chronic disease: HIV and Cancer[J]. AIDS Behav, 2022: 1–11. 10.1007/s10461-022-03638-0 PMC8898333 35249178

[8] Meghan JE, Shannan L, Maoliosa D (2020). Outpatient interventions for managing Acute Complications of Chronic Diseases: a scoping review and implications for patients with CKD[J]. Am J Kidney Dis.

[9] Jiang CH, Zhu F, Qin TT (2020). Relationships between Chronic Diseases and Depression among Middle-aged and Elderly People in China: a prospective study from CHARLS[J]. Curr Med Sci.

[10] Parthasarathy P, Vivekanandan S (2021). An extensive study on the COVID-19 pandemic, an emerging global crisis: risks, transmission, impacts and mitigation[J]. J Infect Public Health.

[11] Sonia, Umair (2021). Umair Waqas Muhammad Faheem.COVID-19 pandemic: stringent measures of Malaysia and implications for other countries[J]. Postgrad Med J.

[12] Means AR, Wagner AD, Kern E (2020). Implementation science to Respond to the COVID-19 Pandemic[J]. Front Public Health.

[13] Antonella Sisto F, Vicinanza LL, Campanozzi (2019). Towards a Transversal definition of psychological resilience: a literature Review[J]. Med (Kaunas).

[14] Vanmeter F (2020). Dante Cicchetti. Resilience[J]. Handb Clin Neurol.

[15] Leodoro JL (2021). Psychological resilience, coping behaviours and social support among health care workers during the COVID-19 pandemic: a systematic review of quantitative studies[J]. J Nurs Manag.

[16] Wu C, Liu YP, Ma SC (2021). The mediating roles of coping styles and resilience in the relationship between perceived social support and posttraumatic growth among primary caregivers of schizophrenic patients: a cross-sectional study[J]. BMC Psychiatry.

[17] Wu Y, Yu WZ, Wu XY (2020). Psychological resilience and positive coping styles among chinese undergraduate students: a cross-sectional study[J]. BMC Psycholoy.

[18] Rutten BPF, Hammels C, Geschwind N, Menne-Lothmann C (2013). Resilience in mental health: linking psychological and neurobiological perspectives[J]. Acta Psychiatr Scand.

[19] Parvar SY, Ghamari N, Pezeshkian F (2022). Prevalence of anxiety, depression, stress, and perceived stress and their relation with resilience during the COVID-19 pandemic, a cross-sectional study[J]. Health Sci Rep.

[20] Esmina, Avdibegović (2017). Mevludin Hasanović.The Stigma of Mental illness and Recovery[J]. Psychiatr Danub.

[21] Kimberley R, Monden A, Philippus, Bria Macintyre. The impact of stigma on psychosocial outcomes following spinal cord injury: a cross-sectional analysis of stigma-mediated relationships[J]. Rehabil Psychol 2021, 66(2): 202–12. 10.1037/rep0000371 10.1037/rep0000371 33382334

[22] Peter Zweifel (2021). Mental health: the burden of social stigma[J]. Int J Health Plann Manage.

[23] Rai SS, Syurina EV, Ruth MH, Peters et al. Non-communicable Diseases-Related stigma: a mixed-methods Syst Review[J] Int J Environ Res Public Health 2020,17(18):6657. 10.3390/ijerph17186657 PMC7559120 32932667

[24] Sharon M, Holder ER, Peterson R, Stephens (2019). Stigma in Mental Health at the Macro and Micro levels: implications for Mental Health Consumers and Professionals[J]. Community Ment Health J.

[25] Rebecca M, Puhl MS, Himmelstein JS. Weight stigma and diabetes stigma: implications for weight-related Health Behaviors in adults with type 2 Diabetes[J]. Clin Diabetes,2022,40(1): 51–61. 10.2337/cd20-0071 10.2337/cd20-0071 PMC8865787 35221472

[26] Meghan Seewald, Lisa A, Martin L, Echeverri (2019). Stigma and abortion complications: stories from three continents[J]. Sex Reprod Health Matters.

[27] Daniela K, Schlüter A, Tennant R, Mills (2018). Risk factors for social withdrawal in amyotrophic lateral sclerosis/motor neurone disease[J]. Amyotroph Lateral Scler Frontotemporal Degener.

[28] Joanie Pellet P, Golay A, Nguyen (2019). The relationship between self-stigma and depression among people with schizophrenia-spectrum disorders: a longitudinal study[J]. Psychiatry Res.

[29] Darawan Thapinta K, Srithanaviboonchai P, Uthis (2022). Association between Internalized Stigma and Depression among people living with HIV in Thailand[J]. Int J Environ Res Public Health.

[30] Rosanna Breaux, Melissa R, Dvorsky, Nicholas P, Marsh et al. Prospective impact of COVID-19 on mental health functioning in adolescents with and without ADHD: protective role of emotion regulation abilities[J]. J Child Psychol Psychiatry 2021, 62(9): 1132–9. 10.1111/jcpp.13382 PMC8014657 33543486

[31] Nicole H, Weiss SR, Forkus, Ateka A, Contractor (2020). The interplay of negative and positive emotion dysregulation on mental health outcomes among trauma-exposed community individuals[J]. J Child Psychol Psychiatry.

[32] Emma Vaughan B, Koczwara E, Kemp et al. Exploring emotion regulation as a mediator of the relationship between resilience and distress in cancer[J]. Psychooncology,2019,28(7): 1506–12. 10.1002/pon.5107 10.1002/pon.5107 31087804

[33] Boardman F, Griffiths F, Kokanovic R (2011). Resilience as a response to the stigma of depression: a mixed methods analysis[J]. J Affect Disord.

[34] Silván-Ferrero P, Recio P, Molero F (2020). Psychological quality of life in people with physical disability: the effect of internalized stigma, collective action and Resilience[J]. Int J Environ Res Public Health.

[35] Miguel Ángel Cano FG, Castro M, De La Rosa (2020). Depressive symptoms and resilience among hispanic emerging adults: examining the Moderating Effects of Mindfulness, distress tolerance, emotion regulation, Family Cohesion, and Social Support[J]. Behav Med.

[36] Chen D, Wu JH, Yao ZX et al. Negative association between resilience and event-related potentials evoked by negative emotion[J]. Sci Rep 2018,8(1): 7149. 10.1038/s41598-018-25555-w PMC5940768 29740037

[37] Olga Troynikov CG, Watson N (2018). Sleep environments and sleep physiology: a review[J]. J Therm Biol.

[38] Handan İnönü Köseoğlu (2021). COVID-19 pandemic and sleep disorders: COVID-somnia[J]. Tuberk Toraks.

[39] Milena K, Pavlova V (2019). Latreille Sleep Disorders[J] Am J Med.

[40] Leo Sher (2020). Sleep, resilience and suicide[J]. Sleep Med.

[41] maura regina ribeiro, juliana zangilorami-raimundo, polyana caroline, et al. Association between sleep quality and depression among institutionalized and community older people - Brazilian Western Amazonia[J]. BMC Psychiatry et al. 2021,21(1): 367. 10.1186/s12888-021-03368-y PMC8299579 34301230

[42] Liu JR, Zhu L (2020). Sleep Quality and Self-Control: the mediating roles of positive and negative Affects[J]. Front Psychol.

[43] Zhang L, Bai YL, Cui XB (2022). Negative emotions and brain: negative emotions mediates the association between structural and functional variations in emotional-related brain regions and sleep quality[J]. Sleep Med.

[44] Adriano DS, Targa ID, Benítez A, Moncusí-Moix (2021). Decrease in sleep quality during COVID-19 outbreak[J]. Sleep Breath.

[45] Uzoji Nwanaji-Enwerem EM, Condon S, Conley (2022). Adapting the health stigma and discrimination Framework to understand the association between stigma and sleep deficiency: a systematic review[J]. Sleep Health.

[46] He S, Ke XJ, Wu Y (2022). The stigma of patients with chronic insomnia: a clinical study[J]. BMC Psychiatry.

[47] Miller MD, Paradis CF, Houck PR (1992). Rating chronic medical illness burden in geropsychiatric practice and research: application of the cumulative illness rating Scale[J]. Psychiatry Res.

[48] Kathryn M, Connor, Jonathan RT (2003). Davidson.Development of a new resilience scale: the Connor-Davidson Resilience Scale (CD-RISC)[J]. Depress Anxiety.

[49] Deepa Rao SW, Choi D, Victorson (2009). Measuring stigma across neurological conditions: the development of the stigma scale for chronic illness (SSCI) [J]. Qual Life Res.

[50] Buysse DJ, Reynolds CF, Monk TH (1989). The Pittsburgh Sleep Quality Index: a new instrument for psychiatric practice and research[J]. Psychiatry Res.

[51] Kroenke K, Spitzer RL, Williams JB (2001). The PHQ-9: validity of a brief depression severity measure[J]. J Gen Intern Med.

[52] Kanokporn Thongkhum N, Peungposop. Nanchatsan Sakunpong.A mixed-methods study to develop a Resilience Scale for Thai Elderly with Chronic Diseases and Depression[J]. Depress Res Treat,2022: 3256981. 10.1155/2022/3256981 PMC8783714 35075398

[53] XN LI, HS YU (2021). The mediating role of resilience in the effects of physical exercise on college students’ negative emotions during the COVID-19 epidemic[J]. Sci Rep.

[54] Quyen G, To C, Vandelanotte K, Cope (2022). The association of resilience with depression, anxiety, stress and physical activity during the COVID-19 pandemic[J]. BMC Public Health.

[55] Hatice, Şahin. Fulya Türk. The Impact of Cognitive-Behavioral Group Psycho-Education Program on Psychological Resilience, Irrational Beliefs, and Well-Being[J]. 2021,39(4): 672–694. 10.1007/s10942-021-00392-5 PMC8016428 33824549

[56] Yang H, Lu YH, Gu YH (2022). Death anxiety among advanced cancer patients: a cross-sectional survey[J]. Support Care Cancer.

[57] Peker A. Serkan Cengiz.Covid-19 fear, happiness and stress in adults: the mediating role of psychological resilience and coping with stress[J]. Int J Psychiatry Clin Pract,2021, 12: 1–9. 10.1080/13651501.2021.1937656 10.1080/13651501.2021.1937656 34253128

[58] Robyn Lewis Brown (2015). Perceived stigma among people with chronic health conditions: the influence of age, stressor exposure, and psychosocial resources[J]. Res Aging.

[59] Jazmín M-R, Ortega-Ortega M, Natera G (2016). Subjective experience and resources for coping with stigma in people with a diagnosis of Schizophrenia: an Intersectional Approach[J]. Qual Health Res.

[60] Sonia Mukhtar (2020). Psychological health during the coronavirus disease 2019 pandemic outbreak[J]. Int J Soc Psychiatry.

[61] Chin ED, Deborah Armstrong (2019). Anticipated stigma and healthcare utilization in COPD and neurological disorders[J]. Appl Nurs Res.

[62] Oliver MD, Datta S, Debora R, Baldwin (2017). A sympathetic nervous system evaluation of obesity stigma[J]. PLoS ONE.

[63] Milton AC, Mullan B, Maccann C (2018). An evaluation of communication barriers and facilitators at the time of a mental health diagnosis: a survey of health professional practices[J]. Epidemiol Psychiatr Sci.

[64] Mulasso A, Argiolu L, Roppolo M (2017). Emotion experience and frailty in a sample of italian community-dwelling older adults[J]. Clin Interv Aging.

[65] Jessica M, Blaxton CS, Bergeman, Brenda R, Whitehead (2017). Relationships among Nightly Sleep Quality, daily stress, and daily Affect[J]. J Gerontol B Psychol Sci Soc Sci.

[66] Yaribeygi H, Panahi Y, Sahraei H (2017). The impact of stress on body function: a review[J]. Excli J.

[67] Rae Dong C, Leung, Mackenzie N, Naert et al. Chronic disease stigma, skepticism of the health system, and socio-economic fragility: qualitative assessment of factors impacting receptiveness to group medical visits and microfinance for non-communicable disease care in rural Kenya[J]. 2021, 16(6): e0248496. 10.1371/journal.pone.0248496 PMC8183981 34097700

[68] Lin S, Schie JV, Ditchburn G (2018). Positive and negative emotions: Differential Associations with Sleep Duration and Quality in adolescents [J]. Brain Behav.

[69] Xiao MJ, Huang GQ, Feng L et al. Impact of sleep quality on post-stroke anxiety in stroke patients[J]. 2020,10(12): e01716. 10.1002/brb3.1716 PMC7749555 33140545

